# Overcoming Microbiome-Acquired Gemcitabine Resistance in Pancreatic Ductal Adenocarcinoma

**DOI:** 10.3390/biomedicines12010227

**Published:** 2024-01-19

**Authors:** Inês Mendes, Nuno Vale

**Affiliations:** 1 PerMed Research Group, Center for Health Technology and Services Research (CINTESIS), Rua Doutor Plácido da Costa, 4200-450 Porto, Portugal; inesmendes_2000@hotmail.com; 2 CINTESIS@RISE, Faculty of Medicine, University of Porto, Alameda Professor Hernâni Monteiro, 4200-319 Porto, Portugal; 3 School of Life and Environmental Sciences, University of Trás-os-Montes and Alto Douro (UTAD), Edifício de Geociências, 5000-801 Vila Real, Portugal; 4 Department of Community Medicine, Information and Health Decision Sciences (MEDCIDS), Faculty of Medicine, University of Porto, Rua Doutor Plácido da Costa, 4200-450 Porto, Portugal

**Keywords:** drug resistance, genetic instability, gemcitabine pathways, microbiome-induced resistance, personalized medicine

## Abstract

Gastrointestinal cancers (GICs) are one of the most recurrent diseases in the world. Among all GICs, pancreatic cancer (PC) is one of the deadliest and continues to disrupt people’s lives worldwide. The most frequent pancreatic cancer type is pancreatic ductal adenocarcinoma (PDAC), representing 90 to 95% of all pancreatic malignancies. PC is one of the cancers with the worst prognoses due to its non-specific symptoms that lead to a late diagnosis, but also due to the high resistance it develops to anticancer drugs. Gemcitabine is a standard treatment option for PDAC, however, resistance to this anticancer drug develops very fast. The microbiome was recently classified as a cancer hallmark and has emerged in several studies detailing how it promotes drug resistance. However, this area of study still has seen very little development, and more answers will help in developing personalized medicine. PC is one of the cancers with the highest mortality rates; therefore, it is crucial to explore how the microbiome may mold the response to reference drugs used in PDAC, such as gemcitabine. In this article, we provide a review of what has already been investigated regarding the impact that the microbiome has on the development of PDAC in terms of its effect on the gemcitabine pathway, which may influence the response to gemcitabine. Therapeutic advances in this type of GIC could bring innovative solutions and more effective therapeutic strategies for other types of GIC, such as colorectal cancer (CRC), due to its close relation with the microbiome.

## 1. Introduction

Pancreatic cancer (PC) is in the list of the deadliest types of cancer. Pancreatic neoplasms are a critical and growing global public health problem, mainly due to the fact that epidemiological estimates for PC reveal that it is the seventh major cause of overall cancer mortality in nations with widely developed industries, as well as the third most frequent cause of mortality in the USA [1]. 

Pancreatic ductal adenocarcinoma (PDAC) is notably the most frequent pancreatic neoplastic cancer, being responsible for approximately 90 to 95% of all pancreatic malignancies [2,3]. PDAC is a violent disease with a 5-year overall survival (OS) rate of merely 11% [4]. The latest advances in chemotherapy treatments have enhanced the results of resectable PDAC [5] but are still insufficient. In clinical studies, it has been shown that as soon as patients are diagnosed with PDAC, 80% of them already have the disease in a late stage, making it impossible to perform surgery with therapeutic intent [6]. The last decade has established combination regimens based on gemcitabine as well as fluoropyrimidine as standard chemotherapy treatments for metastatic PC [7]; however, median OS (mOS) rates are still around 1 year [8,9]. In complete discrepancy to other tumor types, several large-scale trials utilizing targeted agents have been ineffective for PDAC [10,11,12]. Therefore, according to this finding, it is clear that we do not fully understand this disease that is increasing in number year after year and that current treatment options reveal to be inefficient. Consequently, it is urgent to study why this is occurring.

The current obstacles in the treatment of PDAC are, for example, the almost nonexistence of methods aiming to screen PDAC and detect its tumors in an initial stage, in addition to the lack of new and more successful therapeutic modalities. A better understanding of the genomics of PDAC, for example of certain subtypes [13,14], has not yet been reflected in the detection of druggable targets [15], apart from for a minor group of PDAC tumors carrying mutations on the BRCA or DNA repair genes [16]. The majority of people diagnosed with PDAC have an advanced stage of cancer described as having a multifactorial intrinsic resistance, as well as a developed resistance, to existing anticancer therapies. This significant resistance to chemotherapy has been attributed to various PDAC characteristics, such as genetic backgrounds, metabolic modifications, and a heterogeneous tumor microenvironment that has dense fibrosis, in addition to a cellular contexture comprising subgroups of cancer-related fibroblasts, immune-suppressive cells, and also a series of bacteria, which all function differently, molding the particular microenvironment of the tumor microbiome. Therefore, current investigations have led to the advent of a recent avenue of investigation that explains the role that the microbiome plays in acquired resistance to gemcitabine. This has been made possible since next-generation sequencing tools have emerged, and their analyses have characterized a particular microbiome in distinct PDAC tumors [17].

The microbiome is described as a specific microbial community that occupies a reasonably well-established habitat that has different physiochemical characteristics. The microbiome, in addition to encompassing the microorganisms that constitute it, encompasses their domain of activity, which engenders precise ecological niches [18]. This current definition of the microbiome has been proposed by specialists with a deep understanding of its various components. In addition to emphasizing its micro-ecosystem, their definition also takes into account its relationships with macro-ecosystems, such as eukaryotic hosts, thus revealing its crucial role in both health and disease. It is worth noting that the microbiome has recently been identified as a key contributor to the hallmarks of cancer. This phenomenon has been referred to as an "enabling feature," as the microbiome’s impact on cancer development and progression is increasingly recognized. Microbes appear to have an active role in cancer development, being notably considered as a nearly independent variable in this equation [19]. Growing data show that apart from mediating cancer induction and growth, the tumor microbiome may also enable the development of resistance to chemotherapeutic drugs [20,21,22].

Investigations of PDAC reveal that resistance to therapy, as we mentioned before, is one of the many reasons why PDAC has low survival rates. Thus, considering the emerging association of the microbiome with resistance to chemotherapeutic agents such as gemcitabine, the present review was conducted to collect the latest findings on microbiome-induced resistance to gemcitabine. A search was carried out in PubMed and B-ON, and for this, we used “Altered Microbiome” and “PDAC” as keywords jointly or alongside one another, for example, “Antibiotic Combination”, “Gemcitabine Resistance”, or “Personalized Medicine”. Among the obtained data, we used English full-text articles published from 2015 and later. However, to obtain more clarification and deeper comprehension, we also analyzed various older articles. Editorials, case reports, abstracts, commentaries, or manuscripts that were published in languages aside from English did not have a place in the present review.

## 2. The Global Health Problem of Cancer

Cancer is the second most common cause of mortality in the world; it was accountable for approximately 10 million fatalities in 2020 [23]. Nowadays, 9/10 of major pharmaceutical companies concentrate on cancer therapies, with breast cancer (BC) in the lead and being the main aim of studying and developing new drugs. The efficacy of pharmaceuticals in reducing the mortality rate of various types of cancers is widely acknowledged. However, the most recent report by the International Agency for Research on Cancer (IARC), Globocan 2020, has revealed a surge in the incidence of cancer worldwide. According to the report, the number of cancer cases is expected to increase from 19.3 million to 30.3 million by the year 2040. The study underscores the importance of continuous research and development of cancer therapies to curb the growing incidence of the disease [24]. 

Cancer is a multifaceted and heterogeneous illness, modulated by a large number of factors: socioeconomic, environmental, population, tissue, cellular, molecular, or genetic. All of these factors are known to develop over time. In confronting this complex global health problem, numerous scientists have attempted to focus their studies on subjacent disease biology and emerging new groundbreaking therapies. Although the conventional “one-size-fits-all” non-precision method of patient care through surgery, chemotherapy, radiotherapy, and immunotherapy has accomplished some curative efficiency, many obstacles still need to be overcome. One example is the relapse of the disease [25], frequently related to drug resistance [26], which allows tumor metastasis [27,28] and ultimately stimulates cancer development [29].

## 3. Personalized Medicine and Oncology

Currently, we are witnessing an emerging change in the oncology therapy model, specifically in personalized/precision medicine. By utilizing this methodology, a patient’s therapeutic schedule is improved according to a broad comprehension of that patient’s individual systems biology [30] concerning health and disease. This encompasses the collection of extensive information going through the patient’s entire medical history, considering genetic, phenotypic, lifestyle, and psychosocial features to establish the most appropriate therapy plan and probable prognosis. This has been greatly simplified by the comprehensive human genome as well as proteome investigation [31,32,33], and, as expected, by parallel technological innovations [34]. Achieving efficient cancer therapy is the most crucial clinical goal. Nevertheless, the clinical reaction to antitumor agents is often heterogeneous, representing a key challenge needing to be understand so that efficient cancer treatment can be developed. Indeed, in personalized medicine, resistance to chemotherapy and targeted cancer treatment is a major obstacle [35,36]. It is urgent to have new clinical trial designs, focusing on the heterogeneity of tumors and patients in a personalized/precision medicine methodology [37].

New sensitive and precise biomarkers and biomarker panels will be determined, and novel drug targets and drugs recognized. Perhaps it is the adoption of personalized medicine that currently presents major obstacles, specifically the difficulty of large volumes of data, and worries in several public areas concerning privacy, ethical duties, and evenhandedness, with an increasing gap in healthcare parity among high- and low-wage countries and, in several circumstances, even distinct ethnic groups [38]. To handle these difficulties, cooperations and coalitions based on personalized medicine bring together international multidisciplinary groups of innovative leaders, scientists, and oncologists from universities and industries (e.g., The Oncology Think Tank (TOTT), WR Worldwide Innovative Networking in Personalized Cancer Medicine and the European Personalized Medicine Association). This is crucial in simplifying real globalization. In this regard, the COVID-19 pandemic has demonstrated how quickly clinical advances can be accomplished, and that conventional obstacles (e.g., information sharing across large pharmaceutical companies) can be broken down [37].

### 3.1. New Technologies, the Microbiome, and Colorectal Cancer

The emergence of a new technological era has enabled notable advancements in science. Numerous investigations into microbial proteomics have established that microorganisms play a significant role in promoting tumor development and metastatic progression. Moreover, there is growing evidence indicating that the microbiome is partly responsible for the initiation of intestinal tumors. In a pioneering study, Verberkmoes et al. [39] employed an untargeted shotgun mass spectrometry-based metaproteomics approach to obtain the first deep proteome measurements of human distal intestinal microbiota. Additionally, proteomic analyses of stool samples from colorectal cancer (CRC) patients have revealed the presence of several microbial proteins [40]. Bosch et al. have recognized possible novel biomarkers for CRC screening using mass spectrometry (MS) analysis of fecal samples. A panel of four protein biomarkers showed sensitivities of 80% and 45% for screening CRC and late-stage adenomas, correspondingly, with a specificity of 95% [41]. A quantitative metaproteomic investigation characterized microbial protein abundance distinctions between stool samples from CRC patients and healthy controls, demonstrating the pathogenesis of CRC and revealing the encouraging potential of metaproteomics in medical diagnostics in the future [42].

The human microbiome shows potential as a promising target for cancer development and therapy. It may be directly oncogenic, through promoting mucosal inflammation or systemic dysregulation, or may influence anticancer immunity/treatment. Microbiome study may be important in tumor diagnosis and therapy [43]. As a groundbreaking tool in biochemical investigation, proteomics can achieve comprehensive protein profiling of the microbiota, in that way detecting possible biomarkers and revealing modified levels of cancer-associated proteins and biological pathways. Fecal proteomics, a non-invasive test, has notable benefits for the detection and authentication of biomarkers for CRC detection [37]. 

#### Gastrointestinal Cancers (GICs) and Microbiome

Gastrointestinal cancers (GICs) are among the most common types of cancers, affecting individuals diagnosed with oral squamous cell carcinoma (OSCC), tongue squamous cell carcinoma (TSCC), esophagus squamous cell carcinoma (ESCC), gastric cancer (GC), colorectal cancer (CRC), and pancreatic cancer (PC) [44,45]. Several factors have been identified as contributing to gastrointestinal carcinogenesis, including *Helicobacter pylori* infection, host genetics, epigenetics, oral and intestinal microbiota, the role of exosomes, bone marrow-derived stem cells, adult stem cells, and environmental factors. Research has shown that the presence of *Helicobacter pylori* infection in the gastrointestinal tract may increase the risk of developing GICs. Additionally, host genetics and epigenetics may play a significant role in the development of GICs. The oral and intestinal microbiota and the role of exosomes have also been linked to the development of GICs. Furthermore, bone marrow-derived stem cells and adult stem cells may contribute to the progression of GICs. Environmental factors, such as diet and exposure to carcinogenic substances, may also increase the risk of developing GICs [46,47,48,49]. Regardless of multiple attempts in several areas, few advances have been accomplished in patient rescue and therapy. Progress in correct treatments is restricted, as the processes that induce the development of gastrointestinal cancers are not known. Thus, discovery and understanding of the processes implicated in gastrointestinal cancers are critical in identifying an appropriate therapeutic approach [50]. 

Numerous neoplasms can be localized within the cavities of the human body, including the respiratory, gastrointestinal, and genitourinary tracts. These anatomical compartments harbor their distinct microbiomes, which include the fecal microbiome. The presence of *Fusobacterium nucleatum* in colorectal tumor tissue is believed to have been transferred from feces in the intestine to the colonic epithelium, which triggers pro-inflammatory and oncogenic pathways [51,52,53,54]. *Fusobacterium nucleatum*, a Gram-negative anaerobic bacterium, expresses the *FadA* gene, which encodes the FadA protein. This adhesion protein enables it to adhere to E-cadherin and invade CRC cells, initiating the beta-catenin-controlled transcription of oncogenes *Myc* and *Cyclin D1*, which enhances cancer cell proliferation. Remarkably, fecal *F. nucleatum* does not appear to cause colitis or inflammation-related colonic carcinogenesis in mouse models [53]. Instead, it recruits tumor-infiltrating immune cells and increases the number of colonic tumors. *F. nucleatum* also stimulates colorectal tumors through the microRNA *miR21* in the same mouse model. Inhibitors of *miR21* halted CRC cell line proliferation and invasion [55]. MicroRNAs have been shown to be differentially expressed in normal and tumor tissue in the colon and associated with the colon microbiome profile, providing another means through which the intestinal epithelium interacts with bacteria [56]. In mice, *Campylobacter jejuni* results in the development of more and larger colorectal tumors than in uninfected mice. However, this outcome can be minimized with the administration of the antibiotic rapamycin [57]. Other species associated with CRC in the intestine include *Escherichia coli* [58], *Bacteroides fragilis* [59], *Streptococcus bovis* [60], and *Enterococcus fecalis* [61]. Enterotoxigenic *Bacteroides fragilis*, in particular, has been shown to stimulate colonic tumors in mice via IL-17 and possibly through Stat3-driven TH-17T cell reaction [62,63].

Cancer is a complex disease and we have not been able to comprehend it completely. One fact that is remarkably important is that there are a lot of connections between its processes, mechanisms of carcinogenesis, and eventually, therapeutic strategies. Therefore, sometimes the therapeutic strategy for a specific cancer can be found in a completely different one. There is no doubt that the microbiome plays a key role in carcinogenesis in the gastrointestinal tract; thus, we aim to analyze the microbiome’s connection with PC, a GIC that has not seen a lot of therapeutic progress. This may help us to find future investigation directions for providing treatment solutions for the other types of GIC.

The field of personalized medicine in oncology holds promise for addressing the current challenges in cancer treatment, particularly in cancers with high mortality rates such as gastrointestinal cancers (GICs). While linking the microbiome to drug resistance remains a complex task, the first step towards understanding microbiome-induced resistance and developing effective intervention strategies involves analyzing drug mechanisms of action in detail and identifying the differences in gene expression responsible for inducing resistance. While the transition from data analysis to clinical practice presents numerous challenges, genetic tools can be used to elucidate the underlying mechanisms and facilitate the development of effective treatment strategies.

## 4. Chemoresistance in Pancreatic Cancer

Chemoresistance is generally categorized into two groups: intrinsic (de novo or innate) resistance and acquired resistance [64]. Generally, intrinsic resistance indicates the case in which chemotherapy is not effective from the beginning of therapy because of the patient’s genetic factors, while acquired resistance merely occurs after some period of exposure of tumor cells to antitumor chemotherapeutic drugs, as a result of genetic or epigenetic modifications in the cancer cells. Regarding acquired resistance, tumor cells may be sensitive to drugs at the initiation of therapy; however, prolonged therapy eventually results in refractoriness to chemotherapy [65]. In this review, we will focus on acquired resistance to gemcitabine.

Gemcitabine and several anticancer drugs are efficient among people diagnosed with advanced and metastatic PC; however, the occurrence of chemoresistance to gemcitabine brutally restricts the efficacy of this drug. There is no doubt that PC cells are more chemo-resistant to gemcitabine compared to other chemotherapy drugs. As the investigation into the impacts of different chemotherapeutics is still in its beginning, most investigations into chemoresistance in late-stage PC aim to study gemcitabine. The processes inherent to the development of resistance to gemcitabine are still not clear. Several transcription factors, for example, enzymes as well as signaling pathways, included in nucleoside metabolism are more or less associated with the occurrence of resistance to gemcitabine [66,67,68].

## 5. Gemcitabine and Its Presentation

Gemcitabine, first utilized because of its antiviral properties [69], has been extensively used as an antitumor chemotherapeutic agent for several solid tumors and, nowadays, in several lymphomas [70]. Gemcitabine has emerged as a standard therapeutic option for locally advanced and metastatic pancreatic cancer since 1997. This follows the revelation by Burris et al. that gemcitabine demonstrated superior overall survival (OS), performance status, and pain control outcomes when compared to fluorouracil (5-FU) [71]. Its impact on survival was reasonably small (5.65 months vs. 4.41 months); however, it is notable that the clinical benefit response (CBR) of gemcitabine was deeper, practically five times greater, in comparison with 5-FU (23.8% vs. 4.8%) [71].

Gemcitabine remains the main chemotherapy drug used for the treatment of PDAC [72,73]. Nevertheless, the response rate is very modest (around 30%), and even inferior in advanced cases [74,75]. The use of gemcitabine increases average survival by two to three months [76], a very poor outcome. Chemoresistance to gemcitabine develops very quickly [77] and is, consequently, the principal factor restricting drug response. Gemcitabine is utilized as monotherapy or in combination with other anticancer drugs [78]. The outcomes in combinatorial therapies are slightly superior compared to monotherapy, but the high toxicity associated with combinatorial regimens means that gemcitabine is utilized alone on many occasions [79].

### Chemical Structure and Properties

**Figure 1:**
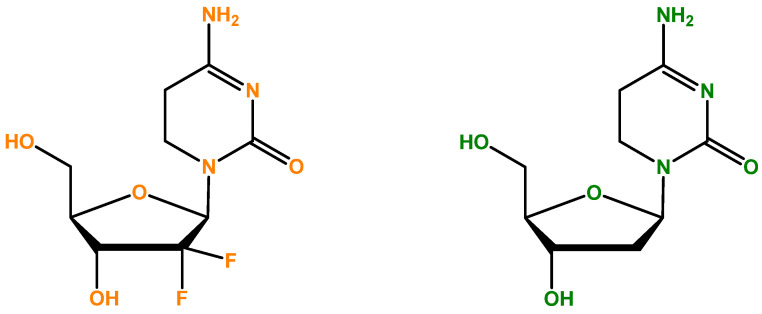
Gemcitabine (dFdC: 2′,2′-difluoro-2′-deoxycytidine)’s chemical formula [84] is represented on the left side. On the right, 2-deoxycytidine (cytosine deoxyribonucleoside) is illustrated, and there is competition between gemcitabine and this nucleoside. It is important to mention that DNA is formed in part by four different nucleosides, and the cytosine deoxyribonucleoside is one of them.

Gemcitabine (also recognized as dFdC: 2′,2′-difluoro-2′-deoxycytidine, Figure 1) is an analog of deoxycytidine nucleoside whose anti-proliferative characteristics are dependent on various inhibitory actions on DNA synthesis, stopping the progression of the cell cycle at the limit of the G1/S phase [80]. Figure 1 illustrates the differentiation that becomes evident when we compare side by side the deoxycytidine nucleoside, which is part of DNA, and gemcitabine, which contains two fluorine atoms. It is clear that the process of DNA synthesis is not capable of differentiating the two molecules; therefore, it randomly incorporates 2-deoxicytidine and gemcitabine [79]. Compared to the cytosine arabinoside (Ara-C), which was the first nucleoside analog shown to be clinically effective, gemcitabine has various distinctive properties and a precise spectrum of activity [81,82]. The unique characteristics of gemcitabine concerning its cellular pharmacology, metabolism, and mechanisms of action occur due to the structural variations between the fluorine substituents in the second position of the furanose ring of dFdC (Figure 1) [83].

## 6. Gemcitabine’s Mechanism of Action

To comprehend the mechanisms of microbiome-induced resistance to gemcitabine treatment, it is essential to analyze its mechanism of action in detail. Consequently, to explore gemcitabine’s mechanism of action, we need to consider three distinct phases of activation as well as one phase of inactivation: how gemcitabine accesses the cell; its intracellular activation; and the impacts of gemcitabine on DNA synthesis and intracellular inactivation [79]. 

### 6.1. Gemcitabine Access to the Cell

After gemcitabine enters into the circulatory system, it transports the drug to the tumor; however, it faces several significant difficulties. The first is the reduced vascular supply of the tumor, and the second, highly significant, is the dense stroma located around the cell, which provides a protective barrier [79]. 

Gemcitabine is highly hydrophilic, therefore passive diffusion across the lipid bilayer of the hydrophobic cellular plasma membrane is not rapid and not significant. To effectively enter into the cells, gemcitabine needs physiologic nucleoside transporter proteins to pass through the plasma membrane [85]. There are two groups of transporter proteins: equilibrative transporters and concentrative transporters. The first group are the bi-directional human equilibrative nuclear transporters (hENTs) and they can be found in almost every cell type, and hENT1, as well as hENT2, can mediate gemcitabine uptake in the direction of the concentration gradient. The second group is the human concentrative nucleoside transporters (hCNTs), which are antiporters that extrude sodium during the import of nucleosides. Human concentrative nucleoside transporter 1 (hCNT1) and hCNT3 are the two recognized concentrative nucleoside/sodium co-transporters in humans, which can transport gemcitabine into a cell using the energy gained from Na+ extrusion. Transporter proteins are capable of functioning against the substrate concentration gradient. However, the human concentrative nucleoside transporter 3 (hCNT3) exhibits a lower level of selectivity compared to the other concentrative transporters in terms of receiving pyrimidine and purine nucleosides. In contrast, hCNT1 and hCNT2 display a more efficient reception of pyrimidine and purine nucleosides, respectively. Despite its broad tissue expression, hCNT3 appears to be less selective in its ability to receive these nucleosides [86,87,88].

**Figure 2:**
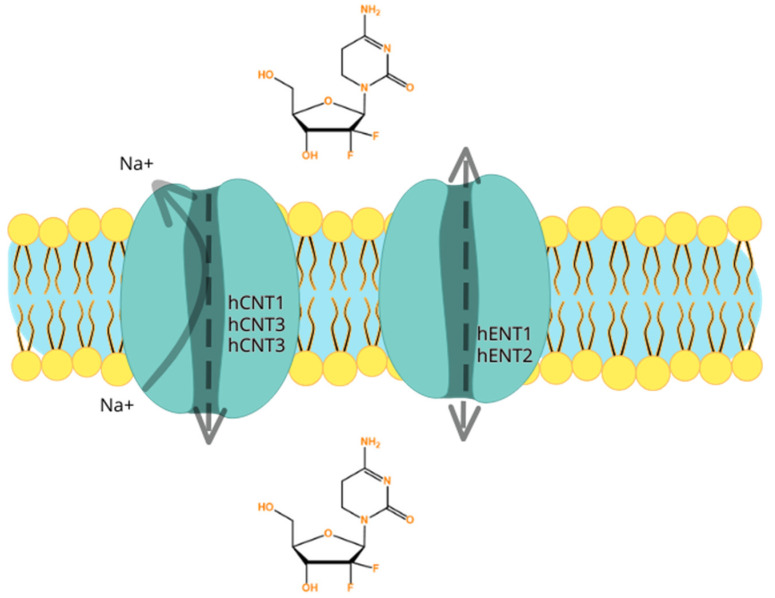
Illustration of the five human nucleoside transporters (NTs) recognized to transport gemcitabine into the cells: hCNT1, hCNT2, hCNT3, hENT1, and hENT2. The hENTs mediate bidirectional transport, while the hCNTs mediate unidirectional transport of gemcitabine into cells. It is important to mention that resistance to gemcitabine may occur firstly because of different expressions of genes that encode for the human nucleoside transporters. If there is low expression, gemcitabine may not be able to enter the cell.

The transport of nucleosides across biological membranes in the direction of the concentration gradient is facilitated by hENTs, which exhibit bidirectional transport capabilities. On the other hand, hCNTs are sodium-dependent symporters that mediate the unidirectional transport of nucleosides into cells [89]. There are five human nucleoside transporters (NTs) known to facilitate the transport of gemcitabine into cells: hCNT1, hCNT2, hCNT3, hENT1, and hENT2. Nonetheless, kinetic studies conducted on human cell lines have revealed that the intracellular uptake of dFdC is primarily mediated by hENT1 (SLC29A1) and, to a lesser extent, by hENT2 (SLC29A2), hCNT1 (SLC28A1), and hCNT3 (SLC28A3) [83,90]. As a result, patients who express or overexpress genes encoding these proteins are expected to exhibit improved intracellular drug access [79]. Patients with reduced levels of the hENT1 protein may exhibit resistance to gemcitabine, which can be clinically verified [91]. Greenhalf et al. [92] conducted a study to investigate changes in overall survival (OS) between patients who underwent ablative surgery, comparing those with high and low hENT1 expressions (ESPAC3 trial). The findings of this study, which was conducted on a significant population of 380 patients, indicated that gemcitabine should not be administered to patients with low hENT1 expression (Figure 2) [92].

### 6.2. Gemcitabine’s Intracellular Activation

Gemcitabine, an anticancer drug, is transported by nucleoside transporters (NTs) and upon entering the cell, it undergoes several phosphorylation steps. Initially, deoxycytidine kinase (dCK) phosphorylates gemcitabine to gemcitabine monophosphate (dFdCMP), which is then further phosphorylated to gemcitabine diphosphate (dFdCDP) by pyrimidine nucleoside monophosphate kinase (NMPK, also known as UMP/CMP), and finally to gemcitabine triphosphate (dFdCTP) by nucleoside diphosphate kinase (NDPK) [80,82]. The first phosphorylation step, catalyzed by dCK, is the rate-limiting step in gemcitabine activation. Downregulation of dCK, which results in acquired resistance to gemcitabine chemotherapy, inhibits the first step of gemcitabine activation. Studies have shown that a low expression of dCK has been linked to poor prognoses and reduced survival rates in resectable pancreatic cancer patients receiving gemcitabine therapy [93,94]. Therefore, monitoring the expression of dCK could be crucial for predicting the responses of pancreatic cancer patients to gemcitabine therapy.

**Figure 3:**
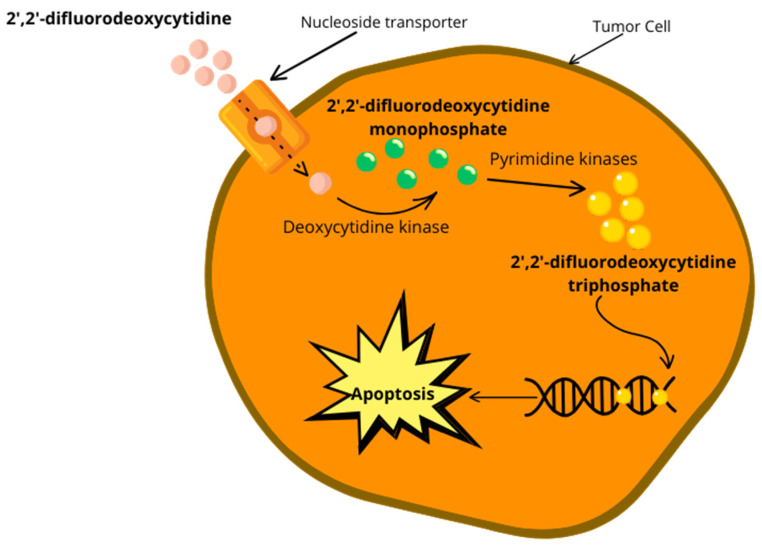
Overview of gemcitabine activation inside the cell and the inhibition of DNA replication, leading to apoptosis.

Gemcitabine is an anticancer drug that aims to block the cell cycle, and to do so, it needs to interfere with DNA replication. To do this, gemcitabine’s major cellular metabolite and active form, dFdCTP, behaves as a competitive substrate of deoxycytidine triphosphate (dCTP). Throughout the DNA replication process, dFdCTP is incorporated into DNA, therefore stopping chain elongation of the DNA and triggering cell death via apoptosis (Figure 3) [80,82].

### 6.3. Effects on DNA Synthesis

dFdC shows cell phase specificity, mainly destroying cells undergoing DNA synthesis. The various impacts on DNA synthesis resulting from the use of gemcitabine may be the reason for the drug’s cytotoxic activity. As we mentioned before, gemcitabine is phosphorylated to gemcitabine monophosphate (dFdCMP) by the enzyme deoxycytidine kinase (dCK) once there is an influx of nucleoside transporters in the cell membranes (Figure 3), which undergo complex intracellular conversion into gemcitabine diphosphate (dFdCDP) as well as triphosphate (dFdCTP), the two active forms. The combined action of these two metabolites is the reason for gemcitabine’s cytotoxicity. On one hand, DNA polymerase is inhibited by competition between dFdCTP and deoxycytidine triphosphate (dCTP). On the other hand, the efficient ribonucleoside reductase inhibitor, dFdCDP, may result in a reduction in deoxyribonucleotide pools, and these are necessary for DNA synthesis, allowing the improvement of dFdCTP action. Essentially, dFdCTP competes with natural deoxycytidine 5′-triphosphate (dCTP) for insertion into replicating DNA; once a dFdCTP molecule is inserted into DNA, an extra deoxyribonucleotide is incorporated into the growing DNA strands, after which DNA synthesis stops and cannot continue anymore, resulting in DNA chain termination. Therefore, this “masked chain termination” mechanism can camouflage dFdCTP and DNA repair enzymes cannot detect it; therefore, gemcitabine ends up locked into the DNA. This has consequences and results in the malfunction of DNA repair mechanisms [83]. 

Gemcitabine has a noteworthy process that enhances its activation, designated “self-potentiation”. Thus, this “self-potentiation” extends the preservation of high concentrations of intracellular gemcitabine metabolites and increases it. Furthermore, it improves the chance of its effective incorporation into nucleic acids, predominantly in DNA, by decreasing the levels of competing natural metabolites [95]. The metabolite of gemcitabine, known as dFdCDP, is highly effective in putting a stop to the action of ribonucleotide reductase (RR), which is an enzyme that plays a crucial role in regulating the biosynthesis of DNA through the control of nucleoside triphosphate (NTP) formation. RR plays a key role in the formation of deoxyribonucleotides, which are essential for DNA synthesis and repair. It facilitates the conversion of cytidinediphosphate (CDP) into deoxycytidindiphosphate (dCDP). If RR is inhibited, it can lead to a decrease in the cellular concentration of the four DNA monomers, which are needed for DNA synthesis, resulting in the simplification of incorporating dFdCTP into DNA [83,95,96]. Gemcitabine, a nucleoside antimetabolite, leads to faster phosphorylation of dFdC in the two active forms, reduced metabolic clearance of gemcitabine nucleotides by deoxycytidine monophosphate deaminase, and better dFdCTP incorporation into DNA. This self-potentiation process is believed to be responsible for the superior antitumor efficiency of dFdC compared to other nucleoside antimetabolites [97,98,99].

Recent research has revealed a novel process of action associated with the primary catabolite of dFdC. Specifically, this process involves the inhibition of thymidylate synthase (TS). The inhibition of this enzyme has been shown to increase the incorporation of 2-deoxyuridine (UdR) into DNA, resulting in indirect harm to the DNA. Additionally, it has been observed that 2′,2′-difluoro-2′-deoxyuridine (dFdU), which was previously considered an inert metabolite of gemcitabine (dFdC), also plays a role in this process. These findings have significant implications for the clinical administration of gemcitabine, whether administered alone or in combination with other treatments such as radiation. Therefore, it can be concluded that thymidylate synthase is a target of dFdC [100].

Gemcitabine has four effects that work to stop cell cycle progression in the synthetic phase. Firstly, it inhibits ribonucleotide reductase (RR), which is associated with the synthesis of deoxycytidine monophosphate that eventually incorporates into DNA. Secondly, it acts as an antimetabolite and blocks the replication process by integrating into the DNA chain. Thirdly, gemcitabine indirectly leads to apoptosis as it is not capable of excision repair. Lastly, this drug also has inhibitory impacts on thymidylate synthase [79]. These mechanisms of gemcitabine could be a starting point to inspire scientists to find innovative ways to fight cancer. 

### 6.4. Intracellular Inactivation

One of the primary mechanisms of gemcitabine’s inactivation is through its conversion to the major metabolite 2′,2′-difluoro-2′-deoxyuridine (dFdU) by cytidine deaminase (CDA) [101]. Cytidine deaminase catabolizes gemcitabine in tissues, transforming gemcitabine into 2′,2′-difluorodeoxyuridine, which then competes with the uptake of gemcitabine due to its transport via the nucleoside transporters hENT and hCNT [102]. This reveals that cytidine deaminase plays a dual role in gemcitabine resistance: one in inactivating gemcitabine and a second in indirectly reducing its transport into the cell. Notably, cytidine deaminase is inhibited if difluorouridine expulsion is blocked; therefore, it has inhibitory effects on cytidine deaminase [103].

## 7. The Pancreatic Human Microbiome

Traditionally, the presence of a microbiome within the pancreas has not been acknowledged. However, Li et al. conducted gene-specific PCR of bacterial 16S rRNA to investigate the microbial components in pancreatic cyst fluids. Their findings revealed predominant microbial constituents such as *Bacteroides*, *Escherichia/Shigella*, and *Acidaminococcus* [104]. These results unveiled the presence of local microbiota in the pancreas and confirmed that pancreatic cyst fluid is a crucial sample for microbial identification. Presently, apart from *P. gingivalis* and *H. pylori*, other microbes have been identified in pancreatic cancer (PC) tissues. Utilizing 16S rRNA gene sequencing, Pushalkar et al. detected high proportions of *Proteobacteria* (45%), *Bacteroidetes* (31%), and *Firmicutes* (22%) species in PC tissues [105]. Interestingly, they concluded that the microbiome proportions in PC tissue differed from those of normal pancreatic tissue. Other research studies have reported similar results [106,107]. Thus, the pancreas is not sterile and possesses a microbial environment that could potentially affect the manifestation and progression of PC. Several multifaceted processes involving numerous factors may alter the pancreatic microbiome. These modifications may occur naturally or through non-natural means [108]. 

### Fungi and Viruses in PC Development

Recent investigations have suggested an association between fungi and viruses and the onset of pancreatic cancer. For example, a prospective cohort study carried out in Sweden found that *Candida* infection in the oral cavity was significantly linked to the occurrence of pancreatic cancer [109]. According to a population-based cohort study conducted in Taiwan, individuals infected with *Candida* are at a notably higher risk of developing PC. The study’s results suggest a significant association between *Candida* infection and the likelihood of PC, which has important implications for clinical practice and public health [110]. 

Further investigation is required to establish the connections between fungal infections and pancreatic cancer. The causal association between hepatitis viruses and hepatocellular carcinoma is well documented. However, some studies suggest that hepatitis viruses may also be linked to pancreatic cancer. Katakura et al. reported elevated serum levels of pancreatic enzymes in patients with viral hepatitis, suggesting a potential association [111]. Jin et al. have established a correlation between the hepatitis B virus and chronic pancreatitis [112]. Studies have found a correlation between chronic hepatitis, chronic pancreatitis, and pancreatic cancer (PC). These findings underscore the importance of considering the possible role of viruses in the development of PC. It is essential to recognize that viral infections can have a lasting impact on the health of an individual and, therefore, should not be overlooked as a possible risk factor for PC [108]. 

There has been a recent discovery of a potential association between fungal and viral infections and the onset of pancreatic cancer. It is crucial to conduct further research into this link to gain a better understanding of the underlying mechanisms and to develop targeted prevention and treatment methods. Prioritizing research in this field is important to advance our knowledge of pancreatic cancer and to ultimately save lives. 

## 8. Microbiome and Gemcitabine Resistance

As we mentioned before, chemotherapy continues to be the first line of therapy for PC at every stage; however, the impact of therapy often varies in each patient [113]. Recent investigations have shown that the microbiome plays a critical role in evaluating the effectiveness and side effects of chemotherapy [114,115]. Chemotherapy could also influence the microbiome through various processes [108]. 

Gemcitabine (2′,2′-difluorodeoxycytidine) is a drug used as a conventional chemotherapeutic treatment that is extensively administered as therapy for different cancers, including PC. Nevertheless, bacteria, mainly belonging to *Gammaproteobacteria*, are able to convert gemcitabine to 2′,2′-difluorodeoxyuridine, an inactive form of gemcitabine [113], utilizing cytidine deaminase (CDA) [116,117]. Geller et al. utilized deep sequencing of bacterial 16S rDNA to identify the microbial composition associated with pancreatic tumors. Their findings revealed that the majority of microbes identified belonged to the *γ-proteobacteria* phylum, with predominant representation from species of *Enterobacter* and *Pseudomonas* [113]. These microbes are capable of generating CDA, resulting in degradation of and resistance to gemcitabine [113]. The therapeutic efficacy of chemotherapeutic drugs is often compromised by the presence of the pyrimidine nucleoside phosphorylase (PyNP) generated by mycoplasmas. This is due to the indirect potentiation of the deamination of these drugs [118]. Additionally, PyNP removes the natural pyrimidine nucleosides uridine, 2′-deoxyuridine, and thymidine, which are known to prevent the deamination of gemcitabine [118]. Moreover, in various types of cancer, certain microbes have been found to decrease the effectiveness of gemcitabine. For instance, *Mycoplasma hyorhinis* contamination has been shown to induce resistance to gemcitabine in laboratory cultures [119,120]. Panos et al. conducted a study in which they observed that the activity of gemcitabine decreased when it was incubated with *E. coli* supernatants [121]. It has been suggested that the combination of antibiotics and gemcitabine could provide a new strategy for enhancing chemosensitivity in patients with pancreatic cancer (PC). However, it should be noted that the use of antibiotics is not without potential adverse effects. Iida et al. demonstrated that in cases of lymphoma, colon carcinoma, and melanoma, mice that were treated with antibiotics or were germ-free and subsequently engrafted with tumors did not respond to CpG-oligonucleotide immunotherapy or platinum chemotherapy. Moreover, antibiotic-treated mice exhibited a downregulation of genes associated with antigen presentation and adaptive immune responses, while genes associated with cancer were upregulated [122]. The question of whether antibiotics can be employed in combination therapy schedules for cancer, and which antibiotics are suitable for such an application, remains a subject of future investigation. Further research is required to gain a better understanding of the potential benefits and risks of utilizing antibiotics in combination with cancer treatment.

Microorganisms have the ability to modulate the efficacy of gemcitabine, a chemotherapeutic agent. However, it is noteworthy that this drug may also disrupt the composition of the human microbiome [123]. Chemotherapy has been reported to cause damage to the gastrointestinal mucosa, through direct cytotoxic effects on cells or by inducing alterations in the intestinal microbiome. As such, it is important to consider the potential adverse effects of chemotherapy on the gastrointestinal tract, as this can have significant implications for the overall health and well-being of the patient. It is therefore recommended that patients undergoing chemotherapy be closely monitored for any signs of gastrointestinal toxicity, and appropriate interventions be taken to manage these side effects and promote optimal health outcomes [124,125].

The normal intestinal microbiome of mice comprises two dominant phyla, namely the *Firmicutes* and *Bacteroidetes* species. However, in gemcitabine-treated mice, *Proteobacteria* and *Verrucomicrobia* were found to replace these phyla. This alteration led to intestinal inflammation and stimulated the development of PC [126,127,128,129,130]. Another investigation revealed that gemcitabine therapy stimulated infection by *Clostridium difficile*, which was undetectable in mice in which gemcitabine was not administered [126]. Besides the microbiome itself, some investigations have also discovered that gemcitabine produces considerable alterations in the metabolomic profiles linked to certain microbes [131,132,133,134]. For instance, Panebianco et al. discovered that inosine levels were notably decreased in mice administered gemcitabine; the mice also exhibited jaundice and had higher hypoxanthine levels [126]. Inosine is a naturally occurring metabolite of adenosine, which possesses anti-inflammatory and immunosuppressive properties. It has been observed that Inosine offers protective effects against inflammation induced by lipopolysaccharide (LPS) [134,135]. However, the use of gemcitabine in cancer treatment can lead to damage to the microbiome, resulting in a vicious cycle that promotes tumor growth. Despite the progress made in this field, this microbe–host–drug relationship remains unclear. The biological complexity of this issue continues to pose a significant challenge to personalized medicine [136]. A more in-depth investigation of the role that the microbiome plays in chemotherapy resistance in PC is necessary to improve the prognosis of this disease [108].

## 9. Antibiotics 

The administration of antibiotics either prior to or during gemcitabine-containing treatment regimens has demonstrated improved outcomes in patients with pancreatic cancer (PC) [137,138]. Recently, a retrospective, single-center Japanese investigation reported a positive correlation between late-stage PC patients who received antibiotics and their increased progression-free survival (PFS) and overall survival (OS) rates when compared with those who did not receive antibiotics during systemic combination therapy with gemcitabine and nab-paclitaxel (5.8 vs. 2.7 months and 11.0 vs. 8.4 months, respectively) [139]. Similarly, a recent retrospective cohort study that included 3850 patients with primary metastatic pancreatic ductal adenocarcinoma (PDAC) who received first-line chemotherapy with gemcitabine or fluorouracil found a significant positive association between the receipt of antibiotics and both OS and cancer-specific survival (CSS) rates. Patients who received antibiotics were matched using propensity scores with those who did not receive antibiotics in the month before or after starting first-line chemotherapy. Receipt of antibiotics was associated with an 11% increase in OS (hazard ratio (HR), 0.89; 95% CI, 0.83–0.96; *p* = 0.003) and a 16% increase in CSS (HR, 0.84; 95% CI, 0.77–0.92; *p* < 0.001) among patients who underwent gemcitabine therapy [140]. However, no correlation was found between the receipt of antibiotics and OS (HR, 1.08; 95% CI, 0.90–1.29; *p* = 0.41) or CSS (HR, 1.12; 95% CI, 0.90–1.36; *p* = 0.29) in patients who received fluorouracil [140]. These findings confirm that antibiotics have the potential to modulate bacteria-mediated gemcitabine resistance and enhance PDAC’s response to treatment.

Fluoroquinolones (FQs), for example, levofloxacin and moxifloxacin, have shown to have proapoptotic and antiproliferative impacts [141,142,143,144] with specific data relating to PC [119], although they may still act through modulation of the immune response [144]. Nevertheless, antibiotics have revealed mixed results when linked to cancer treatment [140], in part due to the extensive variability of the precise antibiotics analyzed, the type of cancer, and the multifaceted microbial–immune interaction involved [145]. It has become evident that addressing the aforementioned issues requires the involvement of multidisciplinary teams.

### 9.1. Fluoroquinolones

Quinolones are a groundbreaking class of antibacterial agents that are entirely synthetic and represent a significant advancement in the field of anti-infective therapy. Unlike natural antibiotics, quinolones were not intentionally designed based on pre-existing molecules. The scaffold of quinolones was subsequently fine-tuned to fluoroquinolones (FQs), which have become a vital component of the treatment regimen for bacterial infections caused by both Gram-positive and Gram-negative bacteria. Currently, only a few antimicrobial agents exhibit a broad spectrum of activity against both types of bacteria, making FQs a unique and valuable class of antibiotics. FQs are considered to be privileged antibiotics because of their extensive bactericidal activity, which occurs at clinically achievable doses, their wide therapeutic index, which means they are relatively safe to use, and their relatively low resistance levels. Additionally, FQs are synthetically tractable, meaning that their chemical structure can be modified to improve their efficacy and reduce their toxicity. As a result, FQs have become widely accepted and are extensively used in clinical settings to fight bacterial infections [146,147]. The mechanism of action of these drugs involves their attachment to a specific intracellular target located in the cytosol of bacterial cells. Once attached, they effectively inhibit the activity of topoisomerase II enzymes, namely gyrase as well as Top IV, with a marked selectivity for prokaryotic enzymes. These enzymes are essential for DNA transcription, duplication, repair, and recombination, and, therefore, the process of DNA replication is interrupted [148]. The innate physicochemical properties of fluoroquinolones (FQs) are remarkable, primarily due to their ability to traverse the orthogonal lipid bilayers of Gram-negative bacteria, which is a task that some naturally occurring antibiotics are incapable of achieving. The remarkable success of fluoroquinolones is attributed to their intrinsic capabilities, including their intracellular targeting and appropriate physicochemical properties. These qualities have made them an excellent reference scaffold for developing highly effective antibacterial agents [143].

Fluoroquinolones, a class of antibiotics that includes ciprofloxacin and moxifloxacin, are frequently used to treat bacterial infections of the lungs, including pneumonia. Recent studies have revealed that fluoroquinolones exhibit inhibitory effects against a range of cancers [149,150,151]. Levofloxacin, a second-generation fluoroquinolone, has been identified as a promising agent for breast cancer treatment due to its selective targeting of breast cancer cells over normal breast cells [152].

In a study conducted by Song et al., levofloxacin was evaluated as a potential treatment for lung cancer. The researchers found that levofloxacin induces mitochondrial dysfunction and oxidative damage in lung cancer cells, leading to growth arrest and apoptosis [142]. This effect was observed across multiple lung cancer cell lines, including A549, H3255, NCL-H69, and H460. These results suggest that levofloxacin is effective against lung cancer cells regardless of their cellular origin and genetic pattern. Notably, the inhibitory effects of levofloxacin were also observed in H460 cells, which are resistant to gefitinib due to *EGFR* and *KRAS* mutations [153]. Moreover, the study’s in vivo xenograft lung cancer mouse model confirmed that levofloxacin significantly arrested lung tumor growth by inhibiting proliferation and inducing apoptosis. These findings add to the growing body of evidence supporting the anti-cancer properties of levofloxacin. In addition to breast cancer, lung cancer can now be added to the list of cancers targeted by levofloxacin [142]. The potential for administering different antibiotics and their positive effects extends beyond PC, as revealed by these data. Although there is limited research on the combination of antibiotics with gemcitabine in pancreatic cancer, it presents a valuable opportunity for exploration in the future.

#### Immunomodulatory Properties of Fluoroquinolones

There is mounting evidence to suggest that some antibiotics can be beneficial not only by killing or inhibiting the growth of bacteria, but also indirectly by affecting the immune system [154,155,156]. The first indication of the potential immunomodulatory properties of FQs was published in the late 1980s [157,158]. Although the mechanisms of the antibacterial activity and effects of FQs have been extensively studied in vitro and in vivo, the mechanism underlying their immunomodulatory activities remains unclear. However, some researchers have proposed a possible cascade of intracellular processes that may lead to stimulatory or inhibitory effects on cytokines, chemokines, and other components of the immune system [159,160]. The immune system plays a crucial role in detecting and eliminating invading organisms and pathogens in the body’s tissues. The colonization of epithelial surfaces by bacteria triggers a complex cytokine network and inflammatory responses due to the release of proinflammatory components such as exoenzymes, exotoxins, polysaccharides, lipoteichoic and teichoic acid, peptidoglycan, and DNA fragments. In such a complex environment, the immune system induces inflammation at the site of perturbation to attack and eliminate foreign tissue/cells, leading to the production of chemokines. It is important to note that inflammation is not a disease but a physiological response to a diseased state utilized by the body to combat the disease itself [161,162].

Chemokines are a diverse family of cytokines that play a vital role in our body’s immune response. They act as chemoattractants, guiding immune cells such as lymphocytes to the sites of inflammation or injury. These signaling proteins can be classified into two categories: inflammatory chemokines and homeostatic chemokines. Inflammatory chemokines are produced in response to pro-inflammatory stimuli, such as viruses, lipopolysaccharides (LPSs), tumor necrosis factors (TNFs), or interleukin-1 (IL-1). On the other hand, homeostatic chemokines are secreted constitutively by certain tissues and are responsible for regulating the migration of leukocytes in the absence of any external stimuli. Understanding the function and regulation of chemokines can provide insights into the mechanisms underlying immune responses and help develop therapies for diseases involving the immune system [163,164,165].

FQs have been found to interact with bacterial adherence to and colonization of epithelial surfaces, while also modulating the release of proinflammatory bacterial products [159,160]. In vitro studies have demonstrated a dose-dependent inhibition of IL-1 and TNF-α synthesis by ciprofloxacin, moxifloxacin, levofloxacin, trovafloxacin, and grepafloxacin at therapeutic concentrations in LPS-stimulated monocytes. Interestingly, these same fluoroquinolones were found to super-induce interleukin-2 (IL-2) in vitro [158]. Research has found that ciprofloxacin induces a direct concentration-dependent inhibition of LPS activity [166]. The effects of ciprofloxacin, rufloxacin, difloxacin, tremafloxacin, and trovafloxacin were evaluated in vivo in a preclinical *Bacteroides fragilis* intra-abdominal infection model. The study showed that subtherapeutic doses eliminated the pathogen in 66% of treated animals [167,168,169,170,171,172]. This in vitro testing of FQs against *Bacteroides fragilis* indicates their ineffectiveness, suggesting that their protective effects are not due to antibacterial properties. Rather, these effects are likely due to the modulation of TNF production in vivo. Additionally, interleukin-2 (IL-2), a T-cell growth factor, is an “early expressed” gene that is produced within a few hours of activation, two to three days before cell division initiation. It has been hypothesized that the IL-2 super-inducing activities of quinolones, such as ciprofloxacin, enhance drug uptake during this crucial stage of cell division, ultimately modulating the rate of proliferation. This phenomenon has been observed in the pulsing of phytohemagglutinin-stimulated peripheral blood lymphocytes (PBLs) with ciprofloxacin [158,172,173].

The immunomodulatory properties carry significant and expansive benefits and implications, which are evident in various fields. The ability to regulate the immune system’s response presents a promising potential in treating various disorders and diseases. The immunomodulatory effects are far-reaching, and their potential in enhancing medical treatments cannot be overstated. Inhibiting monokine synthesis, specifically IL-1 and TNF-, could be advantageous in combating septicemia and septic shock where overstimulation of inflammation by LPS is a major virulence factor of hard-to-treat Gram-negative organisms. Additionally, the super-induction of IL-2 synthesis could be relevant in immunocompromised cancer patients who require external assistance for the regulation of cell proliferation [146]. These benefits and implications should be considered in the context of developing treatment strategies for such conditions. 

It has been noted that the immunomodulatory effects of FQs are typically associated with the presence of co-stimulants, stressors, or triggers on cells or experimental animals. However, when administered to healthy volunteers or intact animals, or when exposed to various cells in vitro, FQs alone did not exhibit any significant immunomodulatory effects [146,147]. Therefore, it is necessary to exercise caution in interpreting the potential therapeutic relevance of FQs, as their intrinsic antibacterial activities may be of greater significance.

## 10. Conclusions and Potential Microbiome Role in PDAC Chemotherapeutics

The microbiome is an emerging cancer hallmark that still seems to be not quite understood. Currently, there are many barriers hindering the success of the microbiome in PDAC chemotherapeutic strategies. There is no doubt that personalized medicine can change the course of the cancer disease as we know it today. Through the advent of omics such as genomics, proteomics, metabolomics, phenomics, and transcriptomics, we have the tools to decipher each patient’s unique features. 

As we discover the bacteria and fungi participating in the disposition, action, and toxicity of drugs, we may be able to inactivate/activate them with the aim of improving chemotherapy and reducing cytotoxicity and cancer progression. Nevertheless, this field of investigation is still at an early stage. Choy et al. have revealed concern that experimental models studying the association between the microbiome and chemotherapy resistance may not be generalizable [116]. The microbiome could be a target in future chemotherapeutic methods; however, it is necessary to first elucidate the effect of host, environmental, and local tumor tissue factors on the constitution and role of the microbiome [116,174,175]. There is a necessity to collect and analyze more robust data, particularly for PDAC, on the medical use of chemotherapy drugs co-administered with antibiotics and/or antifungals, evaluating their influence on disease outcomes. As we start to comprehend the host-, environmental-, and tumor-specific impacts on the tumor microenvironment (TME), we can investigate individualized pharmaco-microbiomics and therapy approaches based on biomarkers and patient demographics [176].

Data on cytidine deaminase (CDA) activity modulation mechanisms in PC are almost non-existent. CDA is encoded by a gene known to be highly polymorphic, therefore future investigations could analyze CDA expression, variants, and polymorphisms, as well as clinical trials that could attempt to correlate the effects of genetic changes in the structure of the enzyme that could result in different reactions to treatment.

Furthermore, the role of ribonucleotide reductase (RR) in the mechanism of action of gemcitabine is of significant importance. Therefore, an investigation into the regulation of RR gene expression could prove particularly compelling. Gene upregulation or downregulation may be a contributing factor to the development of acquired resistance to gemcitabine. As such, it would be advantageous to conduct further studies to analyze genomic alterations, methylation, genetic variants, and mRNA expression levels of the RR genes in pancreatic cancer. 

The field of cancer treatment is witnessing a paradigm shift towards personalized medicine based on genetic traits. A thorough analysis of the expression, variants, and polymorphisms of CDA and the regulation of RR gene expression can pave the way for the development of effective cancer treatments. A greater understanding of the correlation between genetic features and resistance to gemcitabine can facilitate a more personalized approach to treatment. By leveraging patient data and optimizing cancer treatment, we can make a significant impact on the lives of individuals affected by pancreatic cancer. Future research in this area could explore the possibility of microbiome-induced polymorphisms and genetic alterations and their potential correlation with drug toxicity and resistance to gemcitabine. A deeper investigation of this interplay between microbiome and genetics could offer valuable insights into personalized medicine and drug development, and thus warrant further attention from the scientific community. 

## Data Availability

Not applicable.

## References

[1] Rawla P., Sunkara T., Gaduputi V. (2019). Epidemiology of Pancreatic Cancer: Global Trends, Etiology and Risk Factors. World J. Oncol..

[2] Haeberle L., Esposito I. (2019). Pathology of pancreatic cancer. Transl. Gastroenterol. Hepatol..

[3] Kleeff J., Korc M., Apte M., La Vecchia C., Johnson C.D., Biankin A.V., Neale R.E., Tempero M., Tuveson D.A., Hruban R.H. (2016). Pancreatic cancer. Nat. Rev. Dis. Primers.

[4] Siegel R.L., Miller K.D., Fuchs H.E., Jemal A. (2022). Cancer statistics, 2022. CA Cancer J. Clin..

[5] Conroy T., Hammel P., Hebbar M., Abdelghani M.B., Wei A.C., Raoul J.L., Choné L., Francois E., Artru P., Biagi J.J. (2018). FOLFIRINOX or Gemcitabine as Adjuvant Therapy for Pancreatic Cancer. N. Engl. J. Med..

[6] Gobbi P.G., Bergonzi M., Comelli M., Villano L., Pozzoli D., Vanoli A., Dionigi P. (2013). The prognostic role of time to diagnosis and presenting symptoms in patients with pancreatic cancer. Cancer Epidemiol..

[7] Sohal D.P.S., Kennedy E.B., Khorana A., Copur M.S., Crane C.H., Garrido-Laguna I., Krishnamurthi S., Moravek C., O’Reilly E.M., Philip P.A. (2018). Metastatic Pancreatic Cancer: ASCO Clinical Practice Guideline Update. J. Clin. Oncol..

[8] Conroy T., Desseigne F., Ychou M., Bouché O., Guimbaud R., Bécouarn Y., Adenis A., Raoul J.L., Gourgou-Bourgade S., de la Fouchardière C. (2011). FOLFIRINOX versus gemcitabine for metastatic pancreatic cancer. N. Engl. J. Med..

[9] Von Hoff D.D., Ervin T., Arena F.P., Chiorean E.G., Infante J., Moore M., Seay T., Tjulandin S.A., Ma W.W., Saleh M.N. (2013). Increased survival in pancreatic cancer with nab-paclitaxel plus gemcitabine. N. Engl. J. Med..

[10] Van Cutsem E., Tempero M.A., Sigal D., Oh D.Y., Fazio N., Macarulla T., Hitre E., Hammel P., Hendifar A.E., Bates S.E. (2020). Randomized Phase III Trial of Pegvorhyaluronidase Alfa with Nab-Paclitaxel Plus Gemcitabine for Patients with Hyaluronan-High Metastatic Pancreatic Adenocarcinoma. J. Clin. Oncol..

[11] Tempero M., Oh D.Y., Tabernero J., Reni M., Van Cutsem E., Hendifar A., Waldschmidt D.T., Starling N., Bachet J.B., Chang H.M. (2021). Ibrutinib in combination with nab-paclitaxel and gemcitabine for first-line treatment of patients with metastatic pancreatic adenocarcinoma: Phase III RESOLVE study. Ann. Oncol..

[12] Hecht J.R., Lonardi S., Bendell J., Sim H.W., Macarulla T., Lopez C.D., Van Cutsem E., Martin A.J.M., Park J.O., Greil R. (2021). Randomized Phase III Study of FOLFOX Alone or with Pegilodecakin as Second-Line Therapy in Patients with Metastatic Pancreatic Cancer That Progressed after Gemcitabine (SEQUOIA). J. Clin. Oncol..

[13] Le Large T.Y., Prado M.M., Krell J., Bijlsma M.F., Meijer L.L., Kazemier G., Frampton A.E., Giovannetti E. (2016). Bioinformatic analysis reveals pancreatic cancer molecular subtypes specific to the tumor and the microenvironment. Expert Rev. Mol. Diagn..

[14] Schreyer D., Neoptolemos J.P., Barry S.T., Bailey P. (2022). Deconstructing Pancreatic Cancer Using Next Generation-Omic Technologies-from Discovery to Knowledge-Guided Platforms for Better Patient Management. Front. Cell Dev. Biol..

[15] Pecoraro C., Faggion B., Balboni B., Carbone D., Peters G.J., Diana P., Assaraf Y.G., Giovannetti E. (2021). GSK3β as a novel promising target to overcome chemoresistance in pancreatic cancer. Drug Resist. Updates.

[16] Macchini M., Centonze F., Peretti U., Orsi G., Militello A.M., Valente M.M., Cascinu S., Reni M. (2021). Treatment opportunities and future perspectives for pancreatic cancer patients with germline BRCA1-2 pathogenic variants. Cancer Treat. Rev..

[17] Capula M., Perán M., Xu G., Donati V., Yee D., Gregori A., Assaraf Y.G., Giovannetti E., Deng D. (2022). Role of drug catabolism, modulation of oncogenic signaling and tumor microenvironment in microbe-mediated pancreatic cancer chemoresistance. Drug Resist. Updates.

[18] Berg G., Rybakova D., Fischer D., Cernava T., Vergès M.C., Charles T., Chen X., Cocolin L., Eversole K., Corral G.H. (2020). Microbiome definition re-visited: Old concepts and new challenges. Microbiome.

[19] Hanahan D. (2022). Hallmarks of Cancer: New Dimensions. Cancer Discov..

[20] Beatty G.L., Werba G., Lyssiotis C.A., Simeone D.M. (2021). The biological underpinnings of therapeutic resistance in pancreatic cancer. Genes Dev..

[21] Mohindroo C., Hasanov M., Rogers J.E., Dong W., Prakash L.R., Baydogan S., Mizrahi J.D., Overman M.J., Varadhachary G.R., Wolff R.A. (2021). Antibiotic use influences outcomes in advanced pancreatic adenocarcinoma patients. Cancer Med..

[22] Nejman D., Livyatan I., Fuks G., Gavert N., Zwang Y., Geller L.T., Rotter-Maskowitz A., Weiser R., Mallel G., Gigi E. (2020). The human tumor microbiome is composed of tumor type-specific intracellular bacteria. Science.

[23] Sung H., Ferlay J., Siegel R.L., Laversanne M., Soerjomataram I., Jemal A., Bray F. (2021). Global cancer statistics 2020: Globocan estimates of incidence and mortality worldwide for 36 cancers in 185 countries. CA Cancer J. Clin..

[24] Siegel R.L., Miller K.D., Fuchs H.E., Jemal A. (2021). Cancer statistics, 2021. CA Cancer J. Clin..

[25] Mitsudomi T., Kosaka T., Endoh H., Horio Y., Hida T., Mori S., Hatooka S., Shinoda M., Takahashi T., Yatabe Y. (2005). Mutations of the epidermal growth factor receptor gene predict prolonged survival after gefitinib treatment in patients with non-small-cell lung cancer with postoperative recurrence. J. Clin. Oncol..

[26] Middleton J., Stover D., Hai T. (2018). Chemotherapy-exacerbated breast cancer metastasis: A paradox explainable by dysregulated adaptive-response. Int. J. Mol. Sci..

[27] Karagiannis G., Pastoriza J., Wang Y., Harney A., Entenberg D., Pignatelli J., Sharma V., Xue E., Cheng E., D’Alfonso T. (2017). Neoadjuvant chemotherapy induces breast cancer metastasis through a tmem-mediated mechanism. Sci. Transl. Med..

[28] Ming H., Li B., Zhou L., Goel A., Huang C. (2021). Long non-coding rnas and cancer metastasis: Molecular basis and therapeutic implications. Biochim. Biophys. Acta Rev. Cancer.

[29] Ren Y., Zhou X., Yang J., Liu X., Zhao X., Wang Q., Han L., Song X., Zhu Z., Tian W. (2015). Ac1mmyr2 impairs high dose paclitaxel-induced tumor metastasis by targeting mir-21/cdk5 axis. Cancer Lett..

[30] Nice E. (2020). The status of proteomics as we enter the 2020s: Towards personalised/precision medicine. Anal. Biochem..

[31] Lander E., Linton L., Birren B., Nusbaum C., Zody M., Baldwin J., Devon K., Dewar K., Doyle M., FitzHugh W. (2001). Initial sequencing and analysis of the human genome. Nature.

[32] Venter J., Adams M., Myers E., Li P., Mural R., Sutton G., Smith H., Yandell M., Evans C., Holt R. (2001). The sequence of the humangenome. Science.

[33] Adhikari S., Nice E., Deutsch E., Lane L., Omenn G., Pennington S., Paik Y., Overall C., Corrales F., Cristea I. (2020). A high-stringency blueprint of the human proteome. Nat. Commun..

[34] Hanahan D., Weinberg R. (2000). The hallmarks of cancer. Cell.

[35] Dietrich S., Ole’s M., Lu J., Sellner L., Anders S., Velten B., Wu B., Hüllein J., da Silva Liberio M., Walther T. (2018). Drug-perturbation-based stratification of blood cancer. J. Clin. Investig..

[36] Li B., Jiang J., Assaraf Y., Xiao H., Chen Z., Huang C. (2020). Surmounting cancer drug resistance: New insights from the perspective of n-methyladenosine rna modification. Drug Resist. Updates.

[37] Su M., Zhang Z., Zhou L., Han C., Huang C., Nice E.C. (2021). Proteomics, Personalized Medicine and Cancer. Cancers.

[38] Madhusoodanan J. (2020). Health-care inequality could deepen with precision oncology. Nature.

[39] Verberkmoes N., Russell A., Shah M., Godzik A., Rosenquist M., Halfvarson J., Lefsrud M., Apajalahti J., Tysk C., Hettich R. (2009). Shotgun metaproteomics of the human distal gut microbiota. ISME J..

[40] Ang C., Rothacker J., Patsiouras H., Gibbs P., Burgess A., Nice E. (2011). Use of multiple reaction monitoring for multiplex analysis of colorectal cancer-associated proteins in human feces. Electrophoresis.

[41] Bosch L., de Wit M., Pham T., Coupé V., Hiemstra A., Piersma S., Oudgenoeg G., Scheffer G., Mongera S., Sive Droste J. (2017). Novel stool-based protein biomarkers for improved colorectal cancer screening: A case-control study. Ann. Intern. Med..

[42] Long S., Yang Y., Shen C., Wang Y., Deng A., Qin Q., Qiao L. (2020). Metaproteomics characterizes human gut microbiome function in colorectal cancer. NPJ Biofilms Microbiomes.

[43] Picardo S., Coburn B., Hansen A. (2019). The microbiome and cancer for clinicians. Crit. Rev. Oncol. Hematol..

[44] Lin M.T., Song H.J., Ding X.Y. (2018). Long non-coding RNAs involved in metastasis of gastric cancer. World J. Gastroenterol..

[45] Siegel R.L., Miller K.D., Jemal A. (2016). Cancer statistics, 2016. CA Cancer J. Clin..

[46] Abdi E., Latifi-Navid S., Sarvestani F.A., Esmailnejad M.H. (2021). Emerging therapeutic targets for gastric cancer from a host-Helicobacter pylori interaction perspective. Expert Opin. Ther. Targets.

[47] Abdi E., Latifi-Navid S., Zahri S., Yazdanbod A., Pourfarzi F. (2019). Risk factors predisposing to cardia gastric adenocarcinoma: Insights and new perspectives. Cancer Med..

[48] Sun W., Ren Y., Lu Z., Zhao X. (2020). The potential roles of exosomes in pancreatic cancer initiation and metastasis. Mol. Cancer.

[49] Uhlenhopp D.J., Then E.O., Sunkara T., Gaduputi V. (2020). Epidemiology of esophageal cancer: Update in global trends, etiology and risk factors. Clin. J. Gastroenterol..

[50] Siegel R.L., Miller K.D., Jemal A. (2017). Cancer Statistics, 2017. CA Cancer J. Clin..

[51] Flanagan L., Schmid J., Ebert M., Soucek P., Kunicka T., Liska V., Bruha J., Neary P., Dezeeuw N., Tommasino M. (2014). Fusobacterium nucleatum associates with stages of colorectal neoplasia development, colorectal cancer and disease outcome. Eur. J. Clin. Microbiol. Infect. Dis..

[52] Rubinstein M.R., Wang X., Liu W., Hao Y., Cai G., Han Y.W. (2013). Fusobacterium nucleatum promotes colorectal carcinogenesis by modulating E-cadherin/β-catenin signaling via its FadA adhesin. Cell Host Microbe.

[53] Kostic A.D., Chun E., Robertson L., Glickman J.N., Gallini C.A., Michaud M., Clancy T.E., Chung D.C., Lochhead P., Hold G.L. (2013). Fusobacterium nucleatum potentiates intestinal tumorigenesis and modulates the tumor-immune microenvironment. Cell Host Microbe.

[54] Zhou Z., Chen J., Yao H., Hu H. (2018). Fusobacterium and Colorectal Cancer. Front. Oncol..

[55] Yang Y., Weng W., Peng J., Hong L., Yang L., Toiyama Y., Gao R., Liu M., Yin M., Pan C. (2017). Fusobacterium nucleatum Increases Proliferation of Colorectal Cancer Cells and Tumor Development in Mice by Activating Toll-Like Receptor 4 Signaling to Nuclear Factor-κB, and Up-regulating Expression of MicroRNA-21. Gastroenterology.

[56] Yuan C., Burns M.B., Subramanian S., Blekhman R. (2018). Interaction between Host MicroRNAs and the Gut Microbiota in Colorectal Cancer. mSystems.

[57] He Z., Gharaibeh R.Z., Newsome R.C., Pope J.L., Dougherty M.W., Tomkovich S., Pons B., Mirey G., Vignard J., Hendrixson D.R. (2019). Campylobacter jejuni promotes colorectal tumorigenesis through the action of cytolethal distending toxin. GUT.

[58] Arthur J.C., Perez-Chanona E., Mühlbauer M., Tomkovich S., Uronis J.M., Fan T.J., Campbell B.J., Abujamel T., Dogan B., Rogers A.B. (2012). Intestinal inflammation targets cancer-inducing activity of the microbiota. Science.

[59] Wang T., Cai G., Qiu Y., Fei N., Zhang M., Pang X., Jia W., Cai S., Zhao L. (2012). Structural segregation of gut microbiota between colorectal cancer patients and healthy volunteers. ISME J..

[60] Krishnan S., Eslick G.D. (2014). Streptococcus bovis infection and colorectal neoplasia: A meta-analysis. Color. Dis..

[61] Balamurugan R., Rajendiran E., George S., Samuel G.V., Ramakrishna B.S. (2008). Real-time polymerase chain reaction quantification of specific butyrate-producing bacteria, Desulfovibrio and Enterococcus faecalis in the feces of patients with colorectal cancer. J. Gastroenterol. Hepatol..

[62] Wu S., Rhee K.J., Albesiano E., Rabizadeh S., Wu X., Yen H.R., Huso D.L., Brancati F.L., Wick E., McAllister F. (2009). A human colonic commensal promotes colon tumorigenesis via activation of T helper type 17 T cell responses. Nat. Med..

[63] Housseau F., Wu S., Wick E.C., Fan H., Wu X., Llosa N.J., Smith K.N., Tam A., Ganguly S., Wanyiri J.W. (2016). Redundant Innate and Adaptive Sources of IL17 Production Drive Colon Tumorigenesis. Cancer Res..

[64] Rueff J., Rodrigues A.S. (2016). Cancer Drug Resistance: A Brief Overview from a Genetic Viewpoint. Methods Mol. Biol..

[65] Szakács G., Paterson J.K., Ludwig J.A., Booth-Genthe C., Gottesman M.M. (2006). Targeting multidrug resistance in cancer. Nat. Rev. Drug Discov..

[66] Ireland L., Santos A., Ahmed M.S., Rainer C., Nielsen S.R., Quaranta V., Weyer-Czernilofsky U., Engle D.D., Perez-Mancera P.A., Coupland S.E. (2016). Chemoresistance in Pancreatic Cancer Is Driven by Stroma-Derived Insulin-Like Growth Factors. Cancer Res..

[67] Shukla S.K., Purohit V., Mehla K., Gunda V., Chaika N.V., Vernucci E., King R.J., Abrego J., Goode G.D., Dasgupta A. (2017). MUC1 and HIF-1alpha Signaling Crosstalk Induces Anabolic Glucose Metabolism to Impart Gemcitabine Resistance to Pancreatic Cancer. Cancer Cell..

[68] Dauer P., Nomura A., Saluja A., Banerjee S. (2017). Microenvironment in determining chemo-resistance in pancreatic cancer: Neighborhood matters. Pancreatology.

[69] Bianchi V., Borella S., Calderazzo F., Ferraro P., Bianchi L.C., Reichard P. (1994). Inhibition of ribonucleotide reductase by 2′-substituted deoxycytidine analogs: Possible application in AIDS treatment. Proc. Natl. Acad. Sci. USA.

[70] Wong A., Soo R.A., Yong W.P., Innocenti F. (2009). Clinical pharmacology and pharmacogenetics of gemcitabine. Drug Metab. Rev..

[71] Burris H.A., Moore M.J., Andersen J., Green M.R., Rothenberg M.L., Modiano M.R., Cripps M.C., Portenoy R.K., Storniolo A.M., Tarassoff P. (1997). Improvements in survival and clinical benefit with gemcitabine as first-line therapy for patients with advanced pancreas cancer: A randomized trial. J. Clin. Oncol..

[72] Manji G.A., Olive K.P., Saenger Y.M., Oberstein P. (2017). Current and Emerging Therapies in Metastatic Pancreatic Cancer. Clin. Cancer Res..

[73] Conroy T., Bachet J.B., Ayav A., Huguet F., Lambert A., Caramella C., Maréchal R., Van Laethem J.L., Ducreux M. (2016). Current standards and new innovative approaches for treatment of pancreatic cancer. Eur. J. Cancer.

[74] Hashimoto K., Ueno H., Ikeda M., Kojima Y., Hagihara A., Kondo S., Morizane C., Okusaka T. (2009). Do recurrent and metastatic pancreatic cancer patients have the same outcomes with gemcitabine treatment?. Oncology.

[75] Andriulli A., Festa V., Botteri E., Valvano M.R., Koch M., Bassi C., Maisonneuve P., Di Sebastiano P. (2011). Neoadjuvant/preoperative gemcitabine for patients with localized pancreatic cancer: A meta-analysis of prospective studies. Ann. Surg. Oncol..

[76] Ahmed A.A., Marchetti C., Ohnmacht S.A., Neidle S. (2020). A G-quadruplex-binding compound shows potent activity in human gemcitabine-resistant pancreatic cancer cells. Sci. Rep..

[77] Amrutkar M., Gladhaug I.P. (2017). Pancreatic cancer chemoresistance to gemcitabine. Cancers.

[78] Zhang X.-W., Ma Y.-X., Sun Y., Cao Y.-B., Li Q., Xu C.-A. (2017). Gemcitabine in combination with a second cytotoxic agent in the first-line treatment of locally advanced or metastatic pancreatic cancer: A systematic review and meta-analysis. Target. Oncol..

[79] Koltai T., Reshkin S.J., Carvalho T.M.A., Di Molfetta D., Greco M.R., Alfarouk K.O., Cardone R.A. (2022). Resistance to Gemcitabine in Pancreatic Ductal Adenocarcinoma: A Physiopathologic and Pharmacologic Review. Cancers.

[80] Huang P., Chubb S., Hertel L.W., Grindey G.B., Plunkett W. (1991). Action of 2′,2′-difluorodeoxycytidine on DNA synthesis. Cancer Res..

[81] Gandhi V., Plunkett W. (1990). Modulatory activity of 20,20-difluorodeoxycytidine on the phosphorylation and cytotoxicity of arabinosyl nucleosides. Cancer Res..

[82] Hertel L.W., Boder G.B., Kroin J.S., Rinzel S.M., Poore G.A., Todd G.C., Grindey G.B. (1990). Evaluation of the antitumor activity of gemcitabine (20,20-difluoro-20-deoxycytidine). Cancer Res..

[83] Mini E., Nobili S., Caciagli B., Landini I., Mazzei T. (2006). Cellular pharmacology of gemcitabine. Ann. Oncol..

[84] O’Neil M.J. (2006). The Merck Index—An Encyclopedia of Chemicals, Drugs, and Biologicals.

[85] Mackey J.R., Baldwin S.A., Young J.D., Cass C.E. (1998). Nucleoside transport and its significance for anticancer drug resistance. Drug Resist. Updates.

[86] Ritzel M.W., Ng A.M., Yao S.Y., Graham K., Loewen S.K., Smith K.M., Ritzel R.G., Mowles D.A., Carpenter P., Chen X.-Z. (2001). Molecular identification and characterization of novel human and mouse concentrative Na^+^-nucleoside cotransporter proteins (hCNT3 and mCNT3) broadly selective for purine and pyrimidine nucleosides (system cib). J. Biol. Chem..

[87] Young J.D., Yao S.Y., Sun L., Cass C.E., Baldwin S.A. (2008). Human equilibrative nucleoside transporter (ENT) family of nucleoside and nucleobase transporter proteins. Xenobiotica.

[88] Ritzel M.W., Ng A.M., Yao S.Y., Graham K., Loewen S.K., Smith K.M., Hyde R.J., Karpinski E., Cass C.E., Baldwin S.A. (2001). Recent molecular advances in studies of the concentrative Na^+^-dependent nucleoside transporter (CNT) family: Identification and characterization of novel human and mouse proteins (hCNT3 and mCNT3) broadly selective for purine and pyrimidine nucleosides (system cib). Mol. Membr. Biol..

[89] Young J.D., Yao S.Y., Baldwin J.M., Cass C.E., Baldwin S.A. (2013). The human concentrative and equilibrative nucleoside transporter families, SLC28 and SLC29. Mol. Asp. Med..

[90] Mackey J.R., Mani R.S., Selner M., Mowles D., Young J.D., Belt J.A., Crawford C.R., Cass C.E. (1998). Functional nucleoside transporters are required for gemcitabine influx and manifestation of toxicity in cancer cell lines. Cancer Res..

[91] Spratlin J.L., Mackey J.R. (2010). Human Equilibrative Nucleoside Transporter 1 (hENT1) in Pancreatic Adenocarcinoma: Towards Individualized Treatment Decisions. Cancers.

[92] Greenhalf W., Ghaneh P., Neoptolemos J.P., Palmer D.H., Cox T.F., Lamb R.F., Garner E., Campbell F., Mackey J.R., Costello E. (2014). Pancreatic cancer hENT1 expression and survival from gemcitabine in patients from the ESPAC-3 trial. J. Natl. Cancer Inst..

[93] Ohhashi S., Ohuchida K., Mizumoto K., Fujita H., Egami T., Yu J., Tanaka M. (2008). Down-regulation of deoxycytidine kinase enhances acquired resistance to gemcitabine in pancreatic cancer. Anticancer Res..

[94] Sierzega M., Pach R., Kulig P., Legutko J., Kulig J. (2017). Prognostic implications of expression profiling for gemcitabine-related genes (hENT1, dCK, RRM1, RRM2) in patients with resectable pancreatic adenocarcinoma receiving adjuvant chemotherapy. Pancreas.

[95] Plunkett W., Huang P., Xu Y.Z., Heinemann V., Grunewald R., Gandhi V. (1995). Gemcitabine: Metabolism, mechanisms of action, and self-potentiation. Semin. Oncol..

[96] Heinemann V., Xu Y.Z., Chubb S., Sen A., Hertel L.W., Grindey G.B., Plunkett W. (1992). Cellular elimination of 2′,2′-difluorodeoxycytidine 5’-triphosphate: A mechanism of self-potentiation. Cancer Res..

[97] Plunkett W., Huang P., Gandhi V. (1995). Preclinical characteristics of gemcitabine. Anticancer Drugs.

[98] Plunkett W., Huang P., Searcy C.E., Gandhi V. (1996). Gemcitabine: Preclinical pharmacology and mechanisms of action. Semin. Oncol..

[99] Pereira S., Fernandes P.A., Ramos M.J. (2004). Mechanism for ribonucleotide reductase inactivation by the anticancer drug gemcitabine. J. Comput. Chem..

[100] Honeywell R.J., Ruiz V.W., Veerman G., Smid K., Peters G.J. (2015). Inhibition of thymidylate synthase by 2′,2′-difluoro-2′-deoxycytidine (Gemcitabine) and its metabolite 2′,2′-difluoro-2′-deoxyuridine. Int. J. Biochem. Cell Biol..

[101] Heinemann V., Hertel L.W., Grindey G.B., Plunkett W. (1988). Comparison of the cellular pharmacokinetics and toxicity of 2′,2′-difluorodeoxycytidine and 1-beta-D-arabinofuranosylcytosine. Cancer Res..

[102] Hodge L., Taub M., Tracy T. (2011). Effect of its deaminated metabolite, 2′,2′-difluorodeoxyuridine, on the transport and toxicity of gemcitabine in HeLa cells. Biochem. Pharmacol..

[103] Rudin D., Li L., Niu N., Kalari K.R., Gilbert J.A., Ames M.M., Wang L. (2011). Gemcitabine cytotoxicity: Interaction of efflux and deamination. J. Drug Metab. Toxicol..

[104] Li S., Fuhler G.M., Bn N., Jose T., Bruno M.J., Peppelenbosch M.P., Konstantinov S.R. (2017). Pancreatic cyst fluid harbors a unique microbiome. Microbiome.

[105] Pushalkar S., Hundeyin M., Daley D., Zambirinis C.P., Kurz E., Mishra A., Mohan N., Aykut B., Usyk M., Torres L.E. (2018). The Pancreatic Cancer Microbiome Promotes Oncogenesis by Induction of Innate and Adaptive Immune Suppression. Cancer Discov..

[106] Del Castillo E., Meier R., Chung M., Koestler D.C., Chen T., Paster B.J., Charpentier K.P., Kelsey K.T., Izard J., Michaud D.S. (2019). The Microbiomes of Pancreatic and Duodenum Tissue Overlap and Are Highly Subject Specific but Differ between Pancreatic Cancer and Noncancer Subjects. Cancer Epidemiol. Biomark. Prev..

[107] Swidsinski A., Schlien P., Pernthaler A., Gottschalk U., Bärlehner E., Decker G., Swidsinski S., Strassburg J., Loening-Baucke V., Hoffmann U. (2005). Bacterial biofilm within diseased pancreatic and biliary tracts. GUT.

[108] Wang Y., Yang G., You L., Yang J., Feng M., Qiu J., Zhao F., Liu Y., Cao Z., Zheng L. (2019). Role of the microbiome in occurrence, development and treatment of pancreatic cancer. Mol. Cancer.

[109] Huang J., Roosaar A., Axéll T., Ye W. (2016). A prospective cohort study on poor oral hygiene and pancreatic cancer risk. Int. J. Cancer.

[110] Chung L.M., Liang J.A., Lin C.L., Sun L.M., Kao C.H. (2017). Cancer risk in patients with candidiasis: A nationwide population-based cohort study. Oncotarget.

[111] Katakura Y., Yotsuyanagi H., Hashizume K., Okuse C., Okuse N., Nishikawa K., Suzuki M., Iino S., Itoh F. (2005). Pancreatic involvement in chronic viral hepatitis. World J. Gastroenterol..

[112] Jin Y., Gao H., Chen H., Wang J., Chen M., Li G., Wang L., Gu J., Tu H. (2013). Identification and impact of hepatitis B virus DNA and antigens in pancreatic cancer tissues and adjacent non-cancerous tissues. Cancer Lett..

[113] Geller L.T., Barzily-Rokni M., Danino T., Jonas O.H., Shental N., Nejman D., Gavert N., Zwang Y., Cooper Z.A., Shee K. (2017). Potential role of intratumor bacteria in mediating tumor resistance to the chemotherapeutic drug gemcitabine. Science.

[114] Nicholson J.K., Holmes E., Wilson I.D. (2005). Gut microorganisms, mammalian metabolism and personalized health care. Nat. Rev. Microbiol..

[115] Li H., He J., Jia W. (2016). The influence of gut microbiota on drug metabolism and toxicity. Expert Opin. Drug Metab. Toxicol..

[116] Choy A.T.F., Carnevale I., Coppola S., Meijer L.L., Kazemier G., Zaura E., Deng D., Giovannetti E. (2018). The microbiome of pancreatic cancer: From molecular diagnostics to new therapeutic approaches to overcome chemoresistance caused by metabolic inactivation of gemcitabine. Expert Rev. Mol. Diagn..

[117] Geller L.T., Straussman R. (2017). Intratumoral bacteria may elicit chemoresistance by metabolizing anticancer agents. Mol. Cell Oncol..

[118] Voorde J.V., Sabuncuoğlu S., Noppen S., Hofer A., Ranjbarian F., Fieuws S., Balzarini J., Liekens S. (2014). Nucleoside-catabolizing enzymes in mycoplasma-infected tumor cell cultures compromise the cytostatic activity of the anticancer drug gemcitabine. J. Biol. Chem..

[119] Wei M.Y., Shi S., Liang C., Meng Q.C., Hua J., Zhang Y.Y., Liu J., Zhang B., Xu J., Yu X.J. (2019). The microbiota and microbiome in pancreatic cancer: More influential than expected. Mol. Cancer.

[120] Olarerin-George A.O., Hogenesch J.B. (2015). Assessing the prevalence of mycoplasma contamination in cell culture via a survey of NCBI’s RNA-seq archive. Nucleic Acids Res..

[121] Lehouritis P., Cummins J., Stanton M., Murphy C.T., McCarthy F.O., Reid G., Urbaniak C., Byrne W.L., Tangney M. (2015). Local bacteria affect the efficacy of chemotherapeutic drugs. Sci. Rep..

[122] Iida N., Dzutsev A., Stewart C.A., Smith L., Bouladoux N., Weingarten R.A., Molina D.A., Salcedo R., Back T., Cramer S. (2013). Commensal bacteria control cancer response to therapy by modulating the tumor microenvironment. Science.

[123] Paci A., Veal G., Bardin C., Levêque D., Widmer N., Beijnen J., Astier A., Chatelut E. (2014). Review of therapeutic drug monitoring of anticancer drugs part 1—Cytotoxics. Eur. J. Cancer.

[124] Zwielehner J., Lassl C., Hippe B., Pointner A., Switzeny O.J., Remely M., Kitzweger E., Ruckser R., Haslberger A.G. (2011). Changes in human fecal microbiota due to chemotherapy analyzed by TaqMan-PCR, 454 sequencing and PCR-DGGE fingerprinting. PLoS ONE.

[125] Bien J., Palagani V., Bozko P. (2013). The intestinal microbiota dysbiosis and Clostridium difficile infection: Is there a relationship with inflammatory bowel disease?. Ther. Adv. Gastroenterol..

[126] Panebianco C., Adamberg K., Jaagura M., Copetti M., Fontana A., Adamberg S., Kolk K., Vilu R., Andriulli A., Pazienza V. (2018). Influence of gemcitabine chemotherapy on the microbiota of pancreatic cancer xenografted mice. Cancer Chemother. Pharmacol..

[127] Eckburg P.B., Bik E.M., Bernstein C.N., Purdom E., Dethlefsen L., Sargent M., Gill S.R., Nelson K.E., Relman D.A. (2005). Diversity of the human intestinal microbial flora. Science.

[128] Turnbaugh P.J., Ley R.E., Mahowald M.A., Magrini V., Mardis E.R., Gordon J.I. (2006). An obesity-associated gut microbiome with increased capacity for energy harvest. Nature.

[129] Ley R.E., Bäckhed F., Turnbaugh P., Lozupone C.A., Knight R.D., Gordon J.I. (2005). Obesity alters gut microbial ecology. Proc. Natl. Acad. Sci. USA.

[130] Ganesh B.P., Klopfleisch R., Loh G., Blaut M. (2013). Commensal Akkermansia muciniphila exacerbates gut inflammation in Salmonella Typhimurium-infected gnotobiotic mice. PLoS ONE.

[131] Montrose D.C., Zhou X.K., McNally E.M., Sue E., Yantiss R.K., Gross S.S., Leve N.D., Karoly E.D., Suen C.S., Ling L. (2016). Celecoxib Alters the Intestinal Microbiota and Metabolome in Association with Reducing Polyp Burden. Cancer Prev. Res..

[132] Forsgård R.A., Marrachelli V.G., Korpela K., Frias R., Collado M.C., Korpela R., Monleon D., Spillmann T., Österlund P. (2017). Chemotherapy-induced gastrointestinal toxicity is associated with changes in serum and urine metabolome and fecal microbiota in male Sprague-Dawley rats. Cancer Chemother. Pharmacol..

[133] Daliri E.B., Wei S., Oh D.H., Lee B.H. (2017). The human microbiome and metabolomics: Current concepts and applications. Crit. Rev. Food Sci. Nutr..

[134] Da Rocha Lapa F., da Silva M.D., de Almeida Cabrini D., Santos A.R. (2012). Anti-inflammatory effects of purine nucleosides, adenosine and inosine, in a mouse model of pleurisy: Evidence for the role of adenosine A2 receptors. Purinergic Signal..

[135] Gomez G., Sitkovsky M.V. (2003). Differential requirement for A2a and A3 adenosine receptors for the protective effect of inosine in vivo. Blood.

[136] Alexander J.L., Wilson I.D., Teare J., Marchesi J.R., Nicholson J.K., Kinross J.M. (2017). Gut microbiota modulation of chemotherapy efficacy and toxicity. Nat. Rev. Gastroenterol. Hepatol..

[137] Sunakawa Y., Arai H., Izawa N., Mizukami T., Horie Y., Doi A., Hirakawa M., Ogura T., Tsuda T., Nakajima T.E. (2018). Antibiotics may enhance the efficacy of gemcitabine treatment for advanced pancreatic cancer. Ann. Oncol..

[138] Imai H., Saijo K., Komine K., Otsuki Y., Ohuchi K., Sato Y., Okita A., Takahashi M., Takahashi S., Shirota H. (2019). Antibiotic therapy augments the efficacy of gemcitabine-containing regimens for advanced cancer: A retrospective study. Cancer Manag. Res..

[139] Nakano S., Komatsu Y., Kawamoto Y., Saito R., Ito K., Nakatsumi H., Yuki S., Sakamoto N. (2020). Association between the use of antibiotics and efficacy of gemcitabine plus nab-paclitaxel in advanced pancreatic cancer. Medicine.

[140] Fulop D.J., Zylberberg H.M., Wu Y.L., Aronson A., Labiner A.J., Wisnivesky J., Cohen D.J., Sigel K.M., Lucas A.L. (2023). Association of Antibiotic Receipt with Survival Among Patients with Metastatic Pancreatic Ductal Adenocarcinoma Receiving Chemotherapy. JAMA Netw. Open.

[141] Beberok A., Rzepka Z., Respondek M., Rok J., Stradowski M., Wrześniok D. (2019). Moxifloxacin as an inducer of apoptosis in melanoma cells: A study at the cellular and molecular level. Toxicol. Vitr..

[142] Song M., Wu H., Wu S., Ge T., Wang G., Zhou Y., Sheng S., Jiang J. (2016). The antibiotic drug levofloxacin inhibits proliferation and induces apoptosis of lung cancer cells through inducing mitochondrial dysfunction and oxidative damage. Biomed. Pharmacother..

[143] Idowu T., Schweizer F. (2017). Ubiquitous nature of fluoroquinolones: The oscillation between antibacterial and anti-cancer activities. Antibiotics.

[144] Yadav V., Sultana S., Yadav J., Saini N. (2012). Gatifloxacin Induces S and G2-Phase Cell Cycle Arrest in Pancreatic Cancer Cells via p21/p27/p53. PLoS ONE.

[145] Papa V., Schepis T., Coppola G., Chiappetta M.F., Del Vecchio L.E., Rozera T., Quero G., Gasbarrini A., Alfieri S., Papa A. (2023). The Role of Microbiota in Pancreatic Cancer. Cancers.

[146] Mitscher L.A. (2005). Bacterial topoisomerase inhibitors: Quinolone and pyridone antibacterial agents. Chem. Rev..

[147] Ball P. (2000). Quinolone generations: Natural history or natural selection?. J. Antimicrob. Chemother..

[148] Aldred K.J., Schwanz H.A., Li G., McPherson S.A., Turnbough C.L., Kerns R.J., Osheroff N. (2013). Overcoming target-mediated quinolone resistance in topoisomerase IV by introducing metal-ion-independent drug-enzyme interactions. ACS Chem. Biol..

[149] Yadav V., Varshney P., Sultana S., Yadav J., Saini N. (2015). Moxifloxacin and ciprofloxacin induces S-phase arrest and augments apoptotic effects of cisplatin in human pancreatic cancer cells via ERK activation. BMC Cancer.

[150] Suresh N., Nagesh H.N., Sekhar K.V., Kumar A., Shirazi A.N., Parang K. (2013). Synthesis of novel ciprofloxacin analogues and evaluation of their anti-proliferative effect on human cancer cell lines. Bioorg. Med. Chem. Lett..

[151] Kiang J.G., Garrison B.R., Smith J.T., Fukumoto R. (2014). Ciprofloxacin as a potential radio-sensitizer to tumor cells and a radio-protectant for normal cells: Differential effects on γ-H2AX formation, p53 phosphorylation, Bcl-2 production, and cell death. Mol. Cell Biochem..

[152] Yu M., Li R., Zhang J. (2016). Repositioning of antibiotic levofloxacin as a mitochondrial biogenesis inhibitor to target breast cancer. Biochem. Biophys. Res. Commun..

[153] Kharbanda A., Rajabi H., Jin C., Alam M., Wong K.K., Kufe D. (2014). MUC1-C confers EMT and KRAS independence in mutant KRAS lung cancer cells. Oncotarget.

[154] Tauber S.C., Nau R. (2008). Immunomodulatory properties of antibiotics. Curr. Mol. Pharmacol..

[155] Kanoh S., Rubin B.K. (2010). Mechanisms of action and clinical application of macrolides as immunomodulatory medications. Clin. Microbiol. Rev..

[156] Guchhait G., Altieri A., Gorityala B., Yang X., Findlay B., Zhanel G.G., Mookherjee N., Schweizer F. (2015). Amphiphilic tobramycins with immunomodulatory properties. Angew. Chem. Int. Ed. Engl..

[157] Roche Y., Fay M., Gougerot-Pocidalo M.A. (1987). Effects of quinolones on interleukin 1 production in vitro by humanmonocytes. Immunopharmacology.

[158] Riesbeck K., Andersson J., Gullberg M., Forsgren A. (1989). Fluorinated 4-quinolones induce hyperproduction of interleukin 2. Proc. Natl. Acad. Sci. USA.

[159] Riesbeck K. (2002). Immunomodulating activity of quinolones: Review. J. Chemother..

[160] Dalhoff A., Shalit I. (2003). Immunomodulatory effects of quinolones. Lancet Infect. Dis..

[161] Nau R., Eiffert H. (2002). Modulation of release of proinflammatory bacterial compounds by antibacterials: Potential impact on course of inflammation and outcome in sepsis and meningitis. Clin. Microbiol. Rev..

[162] Turner M.D., Nedjai B., Hurst T., Pennington D.J. (2014). Cytokines and chemokines: At the crossroads of cell signalling and inflammatory disease. Biochim. Biophys. Acta Mol. Cell Res..

[163] Baggiolini M. (1998). Chemokines and leukocyte traffic. Nature.

[164] Laing K.J., Secombes C.J. (2004). Chemokines. Dev. Comp. Immunol..

[165] Nitsche D., Schulze C., Oesser S., Dalhoff A., Sack M. (1996). Impact of different classes of antimicrobial agents on plasma endotoxin activity. Arch. Surg..

[166] Gollapudi S.V., Chuah S.K., Harvey T., Thadepalli H.D., Thadepalli H. (1993). In vivo effects of rufloxacin and ciprofloxacin on T-cell subsets and tumor necrosis factor production in mice infected with Bacteroides fragilis. Antimicrob. Agents Chemother..

[167] Thadepalli H., Gollapudi S.V., Chuah S.K. (1986). Therapeutic evaluation of difloxacin (A-56619) and A-56620 for experimentally induced Bacteroides fragilis-associated intra-abdominal abscess. Antimicrob. Agents Chemother..

[168] Thadepalli H., Hajji M., Perumal V.K., Chuah S.K., Gollapudi S. (1992). Evaluation of temafloxacin in a rat model of intra-abdominal abscess. J. Antimicrob. Chemother..

[169] Thadepalli H., Reddy U., Chuah S.K., Thadepalli F., Malilay C., Polzer R.J., Hanna N., Esfandiari A., Brown P., Gollapudi S. (1997). In vivo efficacy of trovafloxacin (CP-99,217), a new quinolone, in experimental intra-abdominal abscesses caused by Bacteroides fragilis and Escherichia coli. Antimicrob. Agents Chemother..

[170] Thadepalli H., Chuah S.K., Reddy U., Hanna N., Clark R., Polzer R.J., Gollapudi S. (1997). Efficacy of trovafloxacin for treatment of experimental Bacteroides infection in young and senescent mice. Antimicrob. Agents Chemother..

[171] King A., May J., French G., Phillips I. (2000). Comparative in vitro activity of gemifloxacin. J. Antimicrob. Chemother..

[172] Riesbeck K., Sigvardsson M., Leanderson T., Forsgren A. (1994). Superinduction of cytokine gene transcription by ciprofloxacin. J. Immunol..

[173] Riesbeck K., Forsgren A. (1998). Commentary on ciprofloxacin-dependent superinduction of IL-2 synthesis and thymidine uptake. Transplantation.

[174] McAllister F., Khan M.A.W., Helmink B., Wargo J.A. (2019). The Tumor Microbiome in Pancreatic Cancer: Bacteria and Beyond. Cancer Cell.

[175] McQuade J.L., Daniel C.R., Helmink B.A., Wargo J.A. (2019). Modulating the microbiome to improve therapeutic response in cancer. Lancet Oncol..

[176] Merali N., Chouari T., Kayani K., Rayner C.J., Jiménez J.I., Krell J., Giovannetti E., Bagwan I., Relph K., Rockall T.A. (2022). A Comprehensive Review of the Current and Future Role of the Microbiome in Pancreatic Ductal Adenocarcinoma. Cancers.

